# Neurological outcome of treatment for patients with impending paralysis due to epidural spinal cord compression by metastatic spinal tumor

**DOI:** 10.1186/s13018-019-1348-x

**Published:** 2019-09-03

**Authors:** Masafumi Maseda, Hiroshi Uei, Masahiro Nakahashi, Hirokatsu Sawada, Yasuaki Tokuhashi

**Affiliations:** 1 Department of Orthopaedic Surgery, Nihon University School of Medicine, 30-1 Oyaguchi-kamicho, Itabashi-ku, Tokyo, 173-8610 Japan; 2 Department of Orthopaedic Surgery, Nihon University Hospital, 1-6 Kanda-Surugadai, Chiyoda-ku, Tokyo, 101-8309 Japan

**Keywords:** Metastatic spine tumor, Epidural spinal cord compression, Paralysis, Radiotherapy, Surgery

## Abstract

**Background:**

Therapeutic intervention has recently been actively performed for metastatic spine tumor even though spinal cord paralysis is not clearly observed, but there has been no report in which the degree of spinal cord compression by tumor was taken into consideration for the paralysis-preventing effect of treatment. Thus, we investigated the neurological outcome after treatment of patients with spinal cord compression in a state of impending paralysis.

**Methods:**

A retrospective cohort study. The subjects were 88 patients with epidural spinal cord compression (ESCC) scale 1b or severer compression with American Spinal Injury Association (ASIA) E spinal metastasis. The neurological outcome after the therapeutic intervention was investigated at regular intervals until death. The therapeutic intervention was posterior decompression and stabilization in 18 patients, stabilization without posterior decompression in 15, and radiotherapy in 55 patients (3 groups).

**Results:**

The ASIA aggravation group was comprised of 15 patients, and the severity of paralysis was ASIA A in 3, B in 3, C in 6, and D in 3. Paralysis appeared in 16.7% in the posterior decompression and stabilization group, 13.3% in the posterior stabilization without decompression group, and 18.8% in the radiotherapy group. In the transverse view, the incidence was high in cases with advancement to the intervertebral foramen and circumferential-type advancement, and paralysis developed in more than 20% of ESCC 1c or severer cases. Factors influencing neurological aggravation were investigated, but there was no significant factor.

**Conclusion:**

In ESCC 1b or severer cases with ASIA E spinal metastasis, paralysis aggravated after therapeutic intervention in 16.7% in the posterior decompression and stabilization group, 13.3% in the stabilization without decompression group, and 16.7% in the radiotherapy group. There was no significant factor influencing the development of paralysis.

## Background

Spinal metastasis of cancer often causes spinal cord compression and induces paralysis. Reportedly, spinal cord compression occurs in 5–14% of all cancer cases [1, 2]. Severe paralysis is irreparable. It has been reported that recovery from complete spinal cord paralysis is difficult to achieve, and thus, metastatic tumor-induced spinal cord paralysis should be treated as quickly as possible before it becomes severe [3–5].

The incidence of spinal cord paralysis after radiotherapy for spinal lesions by fractionated irradiation has been reported to be 1.6–1.9% [6, 7]. However, only spinal lesions were investigated in these reports, and there was no description about the degree of spinal cord compression by tumor. Therefore, no study reported whether therapeutic intervention can truly prevent paralysis in patients with a spinal cord-compressing lesion in a state of impending spinal cord paralysis. In this study, the degree of spinal cord compression was quantitatively evaluated and then the neurological outcome of therapeutic intervention in a state in which spinal cord compression is impending but paralysis has not developed was examined.

## Methods

### Patient population

**Fig. 1:**
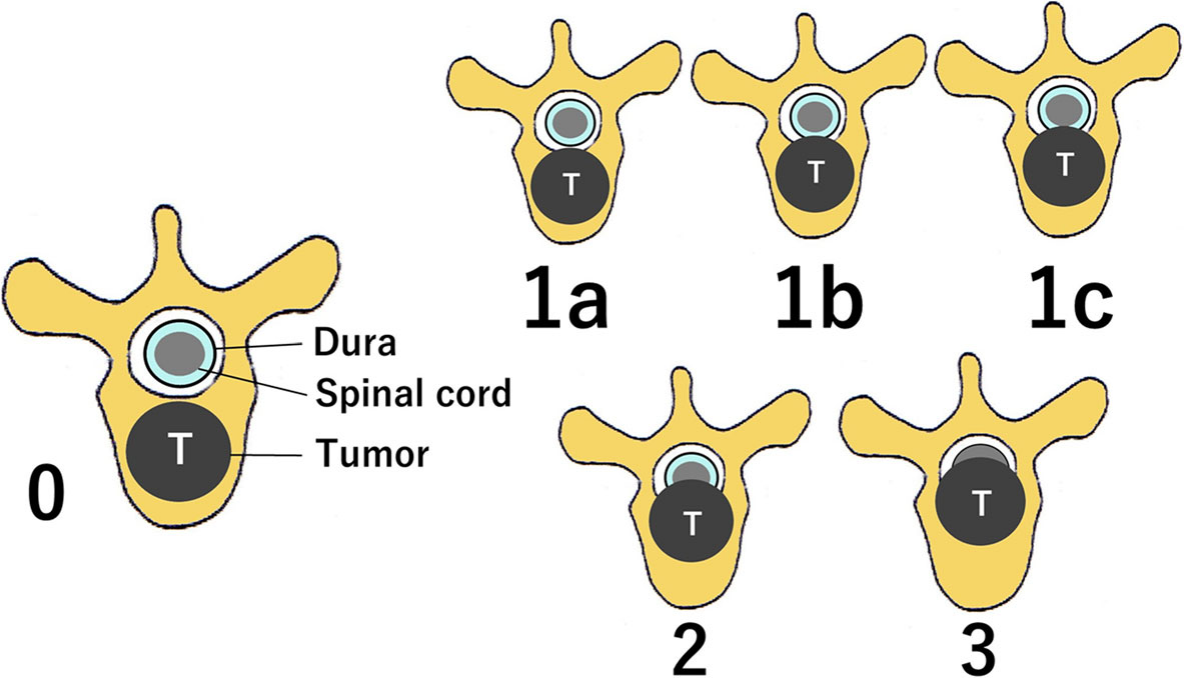
Epidural spinal cord compression (ESCC) scale [8]

This study was a retrospective review of a prospectively collected data from January 1991 to December 2016 at our hospital. The subjects were 88 metastatic spine cancer patients with epidural spinal cord compression (ESCC) scale [8] 1b or severer (Fig. 1) spinal cord compression at American Spinal Injury Association (ASIA) [9] E representing the absence of spinal cord paralysis who received therapeutic intervention. The patients were divided by therapeutic intervention: posterior decompression and stabilization group (*n* = 18), posterior stabilization without decompression group (*n* = 15), and radiotherapy group (*n* = 55) (Table 1). In the posterior decompression and stabilization group, spinal cord decompression at the compressed level and posterior stabilization of 2–3 vertebras with pedicle screws were performed. In the posterior stabilization without decompression group, posterior stabilization with pedicle screws was applied to 2–3 vertebras on the cranial and caudal sides of the compressed level. In the radiotherapy group, 40 Gy radiation was delivered in 2-Gy fractions. In the surgery groups (*n* = 33), adjuvant therapy, such as postoperative radiotherapy, chemotherapy, and treatment with bone-modifying agents, was performed appropriately, and only 3 patients received no adjuvant therapy. In the radiotherapy group, chemotherapy or bone-modifying agent treatment was added to 38 patients.

**Table 1:** Demographic and pre-treatment data according to the treatments employed

Type of treatment	Posterior stabilization with decompression (*n* = 18)	Posterior stabilization without decompression (*n* = 15)	Radiotherapy (*n* = 55)
Male vs. female	12:6	9:6	40:15
Age (years), mean	39–83, 62.5 ± 11.8	40–82, 63.1 ± 13.9	36–85, 65.5 ± 11.5
The level of the spine that exhibited the greatest tumor-related spinal cord compression			
C1-T2 level	5	3	9
T3-L1 level	6	4	22
L2-S1 level	7	8	24
No. of affected vertebral body, mean	1–4, 2.1 ± 1.1	1–12, 3.5 ± 3.4	1–9, 3.3 ± 2.2
Primary site			
Lung	6	5	31
Kidney	3	1	2
Prostate	1	1	4
Liver	3	3	6
Breast	1	3	0
Others	4	2	12
No. of VAS 0	0 (0%)	0 (0%)	6 (10.9%)
Transverse location			
A	3	5	23
AP	2	2	15
AF	9	6	6
APF	4	2	11
ESCC scale			
1b	9	7	28
1c	5	5	19
2	2	3	7
3	2	0	1
Spinal instability neoplastic score	10.1 ± 2.4	10.5 ± 2.5	9.3 ± 2.4
Additional adjuvant therapy	Radiotherapy 8, chemotherapy 11, BMA 3, no 2	Radiotherapy 11, chemotherapy 9, BMA 2, no. 1	Chemotherapy 20, BMA 17, no. 17
Survival period (months), mean	1–116, 16.0 ± 28.0	2–14, 7.2 ± 3.9	0.3–34, 7.7 ± 7.5
Pre-treatment Barthel index, mean	49–100, 81.5 ± 20.7	70–85, 77.5 ± 10.6	10–100, 68.0 ± 30.6

*VAS* visual analog scale score, *A* anterior lesion, *AP* anterior + posterior lesion, *AF* anterior + foraminal lesion, *APF* anterior + posterior + foraminal lesion, *ESCC* epidural spinal cord compression, *BMA* bone-modifying agent

Surgical treatment was prioritized when pain due to spinal instability was severe. However, there was no clear selection criterion of the three treatment methods. It was decided in consideration of the patient’s request and general condition.

### Study measures

For neurological evaluation, the ASIA grade was used. The subjects’ paralysis statuses were assessed at regular intervals until death. The ASIA classification classifies paralysis into the following five categories: A—complete, no sensory or motor function is preserved in the sacral segments S4-S5; B—incomplete, no motor function is preserved below the neurological level, but the sensory function of the sacral segments S4-S5 is preserved; C—incomplete, motor function is preserved below the neurological level, but the muscle strength grade is below 3 in half or more of the key muscles (C5: elbow flexors, C6: wrist extensors, C7: elbow extensors, C8: finger flexors, T1: finger abductor, L2: hip flexors, L3: knee extensors, L4: ankle dorsiflexors, L5: long toe extensors, S1: ankle plantar flexors); D—incomplete, motor function is preserved below the neurological level, and the muscle strength grade is 3 or higher in half or more of the key muscles; E—normal, sensory and motor functions are normal.

### ESCC scale

The ESCC scale is an assessment method proposed by Bilsky et al. in which the degree of compression by tumor is evaluated using a T2-weighted axial view of magnetic resonance imaging (MRI) [8]. The ESCC scale is comprised of six grades: grade 0 represents invasion localized in the bones (bone involvement alone), grade 1 represents epidural compression (epidural impingement), grade 2 represents spinal cord compression with visible cerebrospinal fluid retention (spinal cord compression, but cerebrospinal fluid [CSF] visible), and grade 3 represents severe spinal cord compression without visible CSF (spinal cord compression, but no CSF seen). Grade 1 is further divided into three subgroups: grade 1a represents epidural advancement without deformation of the dural canal (epidural impingement, but no deformation of the thecal sac), grade 1b represents dural canal compression without contact with the spinal cord (deformation of the thecal sac, but without spinal cord abutment), and grade 1c represents dural canal deformities with contact with the spinal cord, but without spinal cord compression (deformation of the thecal sac with spinal cord abutment, but without compression) (Fig. 1).

### Transverse localization of tumors

**Fig. 2:**
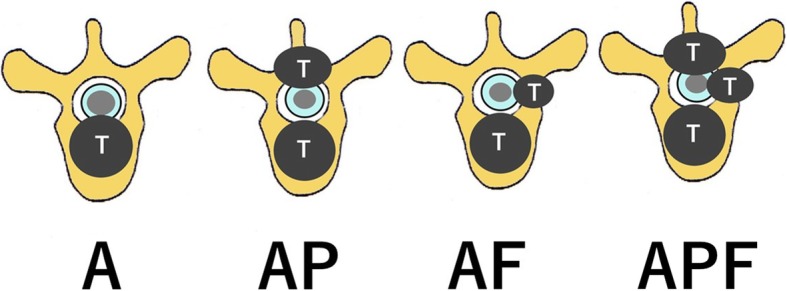
Transverse localization of tumors [10]

The grade of metastatic tumor-induced spinal cord compression can be judged using the ESCC scale, but the ESCC scale does not take the axial localization of the tumor into account. Thus, the following four categories were newly added: (1) anterior compression alone (anterior; A), (2) anterior + posterior compression (anterior + posterior; AP), (3) anterior + foraminal compression (anterior + foraminal; AF), and (4) circumferential compression (anterior + posterior + foraminal; APF) (Fig. 2) [10].

The factors influencing neurological outcomes (sex, age, the maximal spinal cord compression level due to tumor, the primary cancer site, the survival period, the pre-treatment Barthel index, the transverse location of the tumor, the ESCC scale grade, the spinal instability neoplastic score (SINS) [11], and the type of treatment) were analyzed.

### Statistical analysis

For comparisons between two items/groups, the *t* test, Welch’s method, or the Mann-Whitney *U* test was used. For comparisons among multiple items/groups, chi-square test or the Kruskal-Wallis test was used. Statistical analyses were performed using StatMate V® (Atoms Co.; Tokyo, Japan), and *p* values of < 0.05 were regarded as significant.

## Results

### Patients with manifestation of paralysis

**Fig. 3:**
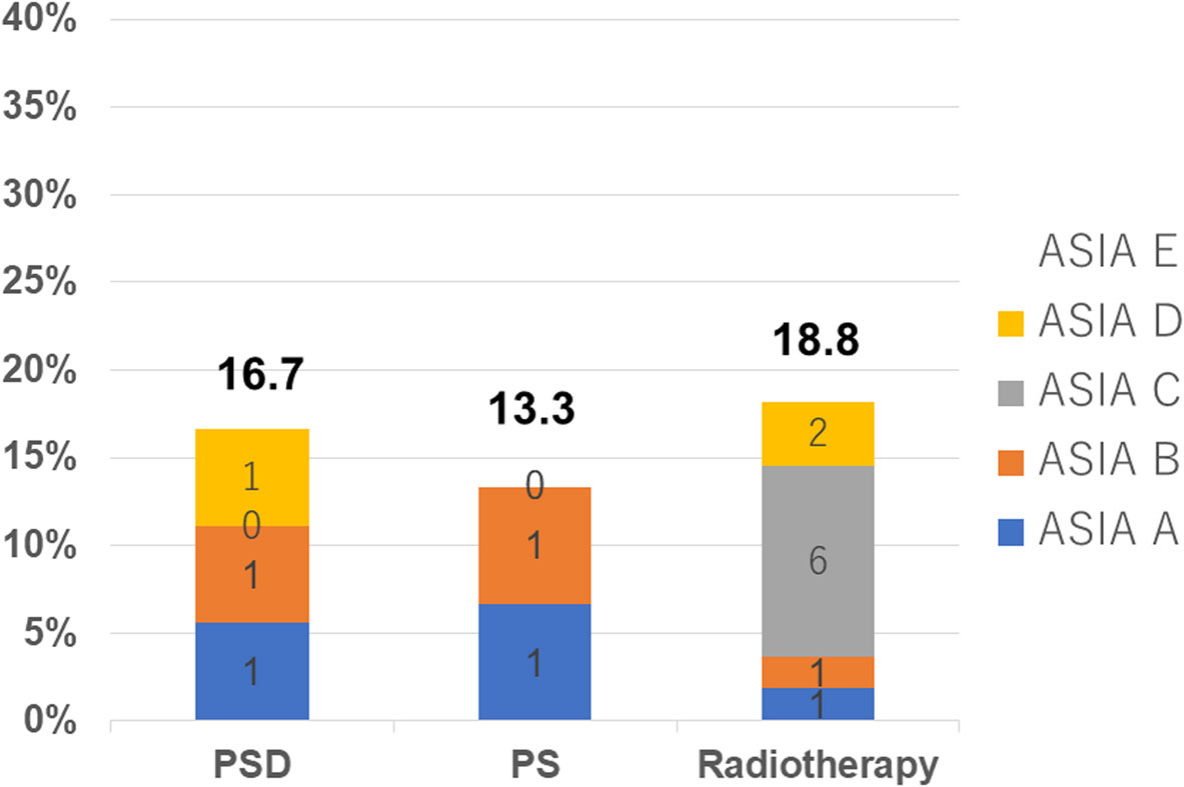
Incidence of paralysis by treatment method

The ASIA aggravation group after therapeutic intervention was comprised of 15 patients, and the grade was A in 3, B in 3, C in 6, and D in 3. Three (16.7%), 2 (13.3%), and 10 (18.8%) patients in the posterior decompression and stabilization, posterior stabilization without decompression, and radiotherapy groups, respectively, were included (Fig. 3). The paralysis onset time was 2 weeks after surgery in some patients, whereas it aggravated 1 year after treatment in others, but most cases aggravated within 3–6 months and paralysis aggravated on follow-up at a regular interval until death.

**Fig. 4:**
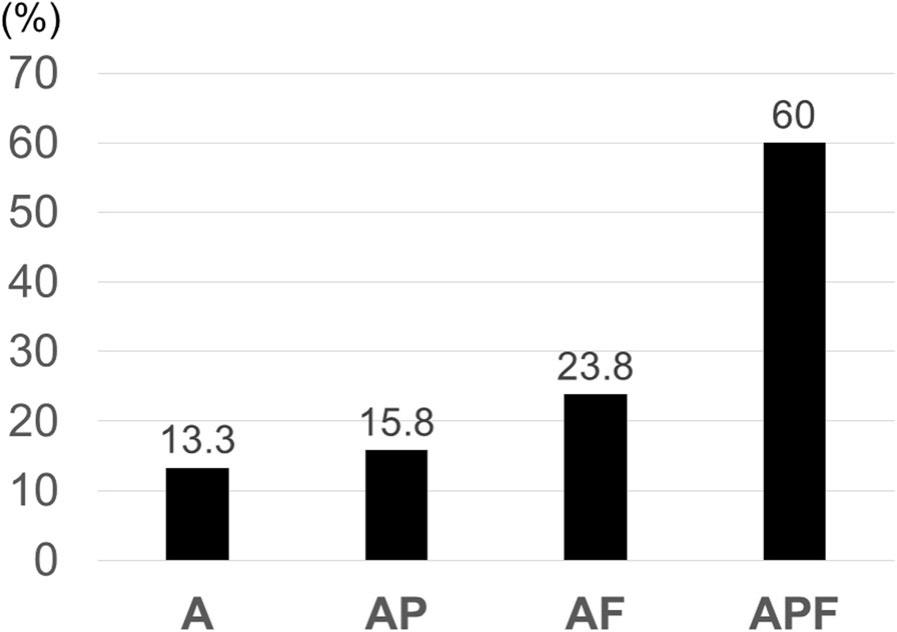
Incidence of paralysis by localization in transverse view

**Fig. 5:**
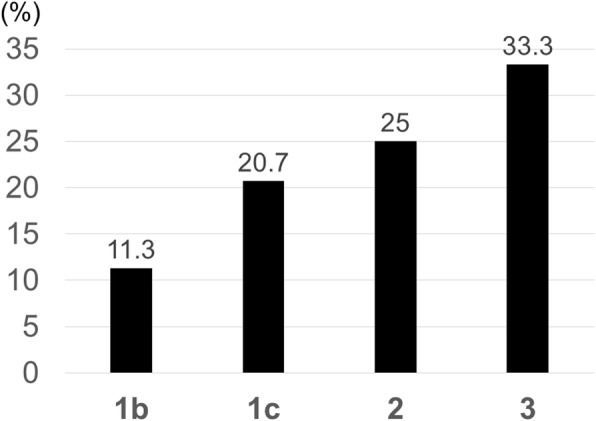
Incidence of paralysis by ESCC scale

In the transverse view, the incidence was higher in cases with advancement to the intervertebral foramen type (transverse type: AF) and circumferential type (transverse type: APF) (23.8% and 60%, respectively) (Fig. 4). Aggravation was noted in more than 20% of cases with ESCC scale 1c or severer compression (Fig. 5).

### The factors influencing neurological outcomes

The sex, the age, the maximal spinal cord compression level due to tumor, the primary cancer site, the survival period, the pre-treatment ASIA classification, the transverse location of the tumor, the ESCC scale grade, the SINS, and the type of treatment were analyzed. However, there was no significant factor influencing neurological outcomes (Table 2).

**Table 2:** Factor influencing neurological outcomes

Factor	Parameter	*p* value
Type of treatment	PSD 3 (16.7%), PS 2 (13.3%), Rad 10 (18.8%)	0.9642
Male vs. female	9/61 (14.8%) vs. 6/27 (22.2%)	0.3902
Age (years), mean	39–85, 64.4 ± 12.6	0.9956
The level of the spine that exhibited the greatest tumor-related spinal cord compression	C1-T2 level 2 (11.8%), T3-L1 level 8 (25.0%), L2-S1 level 5 (12.8%)	0.3539
No. of affected vertebral body, mean	1–8, 3.4 ± 2.3	0.5415
Primary site	Lung 7, kidney 3, colon 1, unknown 3, others 1	0.6713
No. of VAS 0	2 (13.3%)	0.2717
Transverse location	A 6 (13.3%), AP 3 (15.8%), AF 5 (23.8%), APF 3 (60%)	0.9316
ESCC scale	1b 5 (11.3%), 1c 6 (20.7%), 2 3 (25%), 3 1 (33.3%)	0.1325
Spinal instability neoplastic score	10.5 ± 8.6 (vs. 9.5 ± 5.4)	0.2287
Additional adjuvant therapy	No 6/26 (23.1%)	0.3299
Survival period (months), mean	1.5–20, 7.7 ± 6.0	0.4296
Pre-treatment Barthel index, mean	40–100, 89.3 ± 22.4	0.0666

*PSD* posterior stabilization with decompression, *PS* posterior stabilization without decompression, *VAS* visual analog scale score, *A* anterior lesion, *AP* anterior + posterior lesion, *AF* anterior + foraminal lesion, *APF* anterior + posterior + foraminal lesion, *ESCC* epidural spinal cord compression, *No* no additional adjuvant therapy

## Discussion

Early therapeutic intervention has been performed for spinal metastasis with recent prevention of skeletal-related events (SREs). Of SREs (pain requiring radiotherapy, orthopedic surgery, pathological fracture, spinal cord paralysis, and hypercalcemia), the most serious events are spinal cord paralysis and pathological fracture, and to prevent these, surgery and radiotherapy have been performed earlier than before [12, 13].

Prevention of pathological fracture can be easily evaluated based on the pain relief effect after therapeutic intervention, but for the spinal cord paralysis-preventing effect, evaluation of the degree of spinal cord compression and follow-up after treatment are necessary. The degree of spinal cord compression became easily evaluated using the ESCC scale reported by Bilsky et al. as resolution of MRI advanced [8]. This method has been reported to be superior in intra- and inter-rater reproducibility [10]. Thus, using the ESCC scale, a retrospective cohort study of ASIA E patients without paralysis with ESCC 1b or severer spinal cord compression after therapeutic intervention was performed.

Recurrence increased in patients with advancement to the intervertebral foramen, circumferential advancement, or ESCC 1c or severer spinal cord compression, but the difference was not significant. There was also no significant difference in the paralysis-preventing effect among the treatment methods.

It has been reported that direct decompression surgery is more effective than radiotherapy for recovery from spinal cord paralysis [2, 14]. However, no significant difference was noted in the incidence of paralysis among the treatment groups with regard to prevention of paralysis. Regarding the surgical method, it was also clarified that the necessity of decompression is not an absolute factor of paralysis prevention, and there is a limit in additional radiotherapy after surgery as adjuvant therapy.

Quraishi et al. also surveyed functional paralysis outcomes according to the ESCC scale and found that nerve recovery was achieved even when the grade of spinal cord compression was high, i.e., recovery from paralysis can be expected even though the ESCC scale is poor [15]. However, the incidence of paralysis doubled in ESCC 1c cases, suggesting that earlier therapeutic intervention in ESCC 1b is important to improve the treatment outcome.

Regarding the necessity of decompression, because it was not an absolute factor of paralysis prevention, and from viewpoints of low-invasive surgery and early initiation of adjuvant therapy enabled by early wound healing, active introduction of percutaneous pedicle screws was strongly recommended to prevent paralysis [16]. The outcomes of any treatment alone were limited, and multidisciplinary treatment is essential to prevent paralysis [12]. Although there was no significant difference, the incidence of paralysis was much lower in posterior stabilization without decompression group (13.3%) than in radiotherapy group (18.8%). Since posterior stabilization with percutaneous pedicle screws is low invasive and improves ADL early, increasing the possibility of receiving adjuvant therapy, it may be the best surgical method for therapeutic intervention of ASIA E cases with ESCC 1b or severer compression.

Since there is a limit in current normal radiotherapy, it may be important to aim at introducing stereotactic radiosurgery. Recent stereotactic radiosurgery has been reported to significantly prolong the asymptomatic period without allowing local progression [17, 18]. Unfortunately, its application for spinal metastasis is not covered by national health insurance in Japan.

There are several limitations to this study. For neurological evaluation, we did not use electromyography or related studies. Therefore, we cannot deny that measurement of the ASIA grade could be inaccurate. As the number of patients was small, investigation based on the primary tumor origin was not possible, being a limitation of a single-center study. The study was not prospective, and the method could not be limited to one treatment due to the nature of the disease in Japan.

## Conclusions

The incidence of paralysis after therapeutic intervention in 88 ASIA E patients without paralysis with ESCC scale 1b or severer spinal cord compression was 17.0%. There was no significant factor influencing neurological outcomes.

## Data Availability

All data used and analyzed during this study are available from the corresponding author on reasonable request.

## Abbreviations

A: Anterior compression
AF: Anterior + foraminal compression
AP: Anterior + posterior compression
APF: Circumferential compression (anterior + posterior + foraminal compression)
ASIA: American Spinal Injury Association
CSF: Cerebrospinal fluid
ESCC: Epidural spinal cord compression
MRI: Magnetic resonance imaging
SREs: Skeletal-related events
